# Supplementary material for: Patient Education on Exercise Prehabilitation Among Patients Receiving Neoadjuvant Therapy for Cancer Surgery in China: A Mixed-Methods Study
Source: Healthcare (Basel). 2025 Feb 22;13(5):477. doi: 10.3390/healthcare13050477 (PMC11899062; doi:10.3390/healthcare13050477)
Supplement: Supplementary file 1 [file healthcare-13-00477-s001.zip › healthcare-3463205-supplementary.pdf]

Current Practices, Facilitators, and Barriers to Patient Education on Exercise Prehabilitation among Patients Receiving Neoadjuvant Therapy for Cancer Surgery in China: a Mixed-Methods Study

Xiaohan Xu[1,2,†]; Jiao Zhang[1,†]; Yuelun Zhang[3]; Tianxue Yang[4]; Xuerong Yu[1,*]

1 Department of Anesthesiology, Peking Union Medical College Hospital, Chinese Academy of Medical Science and Peking Union Medical College, Beijing 100730, China

2 Department of Anesthesia, Critical Care and Pain Medicine, Center for Anesthesia Research Excellence (CARE), Beth Israel Deaconess Medical Center, Harvard Medical School, Boston, MA 02215, USA

3 Center for Prevention and Early Intervention, National Infrastructures for Translational Medicine, In-stitute of Clinical Medicine, Peking Union Medical College Hospital, Chinese Academy of Medical Science and Peking Union Medical College, Beijing 100730, China

4 School of Sports Medicine and Physical Therapy, Beijing Sport University, Beijing 100084, China

* Correspondence: yxr313@aliyun.com; Tel.: +86-010-6915-2020

† These authors contributed equally to this work.

List of Supplements

Supplements 1: Questionnaire on the Current Practices of Exercise Prehabilitation Before, During, and After Neoadjuvant Therapy for Cancer Surgery

Supplements 2: Interview Guide

Supplements 3: Participant Characteristics

Supplements 4: Quotations

Supplements S1: Questionnaire on the Current Practices of Exercise Prehabilitation Before, During, and After Neoadjuvant Therapy for Cancer Surgery

1. Subject ID ________________________________

2. Height (cm) ________________________________

3. Weight (kg) ________________________________

Before Illness：

4. Did you engage in vigorous-intensity aerobic activity (such as running, jumping rope, hiking uphill, and heavy yard work) before the illness? [Single-choice question]

- Yes
- No (Please skip to Question 6)

5. What was the volume of your vigorous-intensity aerobic activity before illness?

- Minutes per session: ____________
- Sessions per week: ____________

6. Did you engage in moderate-intensity aerobic activity (such as walking, bicycling, and home repair work) before the illness? [Single-choice question]

- Yes
- No (Please skip to Question 8)

7. What was the volume of your moderate-intensity aerobic activity before illness?

- Minutes per session: ____________

- Sessions per week: ____________

8. Did you engage in muscle-strengthening (such as lifting dumbbells using specific muscle groups) activities before the illness? [Single-choice question]

- Yes
- No (Please skip to Question 10)

9. What was the volume of your muscle-strengthening activities before illness?

- Minutes per session: ____________
- Sessions per week: ____________

During Neoadjuvant Therapy:

10. Quality of life during neoadjuvant therapy [Matrix single-choice question] *

Score	0	1	2
Mobility			
Self-care			
Usual Activities			
Pain/Discomfort			
Anxiety/Depression			

After Neoadjuvant Therapy Until Before Surgery:

11. Did you engage in vigorous-intensity aerobic activity after neoadjuvant therapy until before surgery? [Single-choice question]

- Yes

- No (Please skip to Question 13)

12. What was the volume of your vigorous-intensity aerobic activity after neoadjuvant therapy until before surgery?

- Minutes per session: ____________

- Sessions per week: ____________

13. Did you engage in moderate-intensity aerobic activity after neoadjuvant therapy until before surgery? [Single-choice question]

- Yes

- No (Please skip to Question 15)

14. What was the volume of your moderate-intensity aerobic activity after neoadjuvant therapy until before surgery?

- Minutes per session: ____________

- Sessions per week: ____________

15. Did you engage in muscle-strengthening activities after neoadjuvant therapy until before surgery? [Single-choice question]

- Yes

- No (Please skip to Question 17)

16. What was the volume of your muscle-strengthening activities after neoadjuvant therapy until before surgery?

- Minutes per session: ____________

- Sessions per week: ____________

17. Quality of life after neoadjuvant therapy until before surgery:

Score	0	1	2
Mobility			
Self-care			
Usual Activities			
Pain/Discomfort			
Anxiety/Depression			

18. Numeric Rating Scale after neoadjuvant therapy until before surgery:

[0 (no pain) to 10 (worst pain)] _________________________________

Perceived Importance and Knowledge of Prehabilitation:

19. How important do you think the following aspects are for your recovery? [Matrix single-choice question]

Score	1<br>Very unimportant	2<br>Unimportant	3<br>Neutral	4<br>Important	5<br>Very Important
Exercise					
Nutrition					

Psychology

---

20. How much do you know about implementing exercise prehabilitation practice?

- Unaware that I should do exercise before surgery
- Aware that I should do some exercise before surgery, but unsure of the specifics, such as how or how much.
- Have some, but limited, knowledge about the type, volume, and intensity of exercise prehabilitation
- Have a clear and comprehensive knowledge about the type, volume, and intensity of exercise prehabilitation

21. Through which channel(s) did you learn how to implement exercise prehabilitation? [Multiple-choice question]

- Surgeons
- Rehabilitation physicians
- Oncologists
- Anesthesiologists
- Other doctors
- Nurses
- Friends or family
- Media
- Other ____________

22. Which factor(s) do you believe affect your implementation of preoperative exercise?

[Multiple-choice question]

- Physical discomfort prevented me from exercising

- Concerned that exercise may worsen the condition (e.g., bleeding, catching a cold)

- Lack of familiar exercise facilities or groups due to environmental changes

- Busy with medical appointments or treatments, no time

- Feeling depressed, unwilling to exercise

- Other ____________

Supplements S2: Interview Guide

For patients:

1. Where are you from — Beijing or another province?
2. Did you have an exercise routine before your illness? How did this routine influence your exercise practice during and after neoadjuvant therapy?
3. Did you exercise during or after neoadjuvant therapy? Why or why not?
4. Are you aware that you should exercise before surgery? How much do you know the benefits of exercise prehabilitation?
5. Did your surgeon, oncologist, or anesthesiologist recommend exercise, and if so, did they provide guidance on how to do it?
6. Did you see a rehabilitation physician before surgery? If so, when? Was this recommended by someone? Did the rehabilitation physician's assistance prove beneficial?
7. If you have to pay for guidance and supervision of exercise, how much are you willing to spend?
8. If your doctor provides an exercise plan before surgery, how willing are you to follow it? Which doctor would be most persuasive in encouraging you to exercise—your surgeon, oncologist, anesthesiologist, rehabilitation physician, or someone else?
9. If your doctor provides an exercise plan before surgery, what obstacles do you anticipate in implementing it? Do you have any concerns?

For surgeons, oncologists, and anesthesiologists:

1. Are you familiar with exercise prehabilitation? How well do you understand its benefits? Please rank the importance of exercise, nutrition, and psychology in prehabilitation.
2. Do you believe your patients should exercise during or after neoadjuvant therapy? Have you advised them to exercise? Why or why not? If yes, how did you provide this advice?
3. How knowledgeable are you about implementing exercise prehabilitation, including the type, intensity, and volume of exercise? Do you feel the need to learn more about it?
4. Have you provided your patients with guidance or a plan for exercise prehabilitation? If yes, how detailed was it? If not, why?
5. If you provide an exercise prehabilitation plan to your patients, how willing do you anticipate they would be to follow it?
6. What challenges do you think your patients might face when implementing exercise prehabilitation? If your patients need to pay for guidance and supervision, how much do you think they would be willing to spend?
7. Have you referred your patients to rehabilitation physicians? Why or why not? If yes, when did you make the referral?
8. Which doctor do you think is most persuasive in encouraging patients to exercise — a surgeon, oncologist, anesthesiologist, or rehabilitation physician?
9. Which doctor do you believe should be responsible for supervising patients' exercise — a surgeon, oncologist, anesthesiologist, or rehabilitation physician?
10. What challenges do you face in educating patients about exercise prehabilitation?
11. Can you provide any recommendations for improving patient education on exercise prehabilitation?

For rehabilitation physicians and nurses:

1. How many patients scheduled for neoadjuvant therapy come to you for exercise prehabilitation each week? How do they find out that they can seek help from you—are they referred by other doctors?
2. When do patients typically see you for exercise prehabilitation—before, during, or after neoadjuvant therapy?
3. How do you guide and supervise patients in their exercise prehabilitation? Do you use in-person demonstrations? Do we have on-line resources?
4. How do you feel about patients' adherence to your exercise plans? What will you do when patients were unable to follow your recommendations?
5. What challenges do you think patients may encounter when implementing exercise prehabilitation? If they need to pay for guidance and supervision, how much do you think they would be willing to spend?
6. Which doctor do you believe is the most persuasive in encouraging patients to exercise—a surgeon, oncologist, anesthesiologist, or rehabilitation physician?
7. Which doctor do you think should be responsible for supervising patients' exercise—a surgeon, oncologist, anesthesiologist, or rehabilitation physician?
8. What challenges do you face in educating and supervising patients on exercise prehabilitation?
9. Can you offer any recommendations for improving patient education on exercise prehabilitation?

Supplements S3: Participant Characteristics

No.	Role	Sex	Age (year)	Characteristics
1	Patient	M	60-70	• Lived in rural area distant from Beijing<br>• Did labor work before illness
2	Patient	F	50-60	• Lived in Beijing<br>• Maintained a regular exercise habit before illness
3	Patient	M	60-70	• Lived in Beijing<br>• Did not have an exercise habit prior to illness
4	Patient	F	70-80	• Lived in a big city close to Beijing<br>• Depressed after being diagnosed of the illness
5	Relative of Patient 1	F	40-50	• Lived in Beijing<br>• Provided housing and care for patient No.1 during neoadjuvant therapy
6	Thoracic Surgeon	F	50-60	• Vice professor specializing in esophageal cancer<br>• Cooperated with the department of rehabilitation for years
7	Thoracic Surgeon	F	40-50	• Vice professor specializing in lung cancer<br>• Cooperated with the department of rehabilitation for years
8	Gynecological surgeon	M	50-60	• Professor specializing in gynecologic cancer<br>• Amateur athlete
9	Gynecological surgeon	F	50-60	• Professor specializing in gynecologic cancer
10	Gynecological surgeon	F	60-70	• Professor specializing in gynecologic cancer
11	General Surgeon	F	60-70	• Professor specializing in upper digestive tract cancer

				• Expert in ERAS<br>• Expert in nutrition
12	General Surgeon	F	40-50	• Vice professor specializing in lower digestive tract cancer
13	General Surgeon	M	60-70	• Professor specializing in upper digestive tract cancer
14	General Surgeon	M	30-40	• Attending physician specializing in lower digestive tract cancer
15	General Surgeon	M	50-60	• Professor specializing in lower digestive tract cancer<br>• Science popularization expert
16	General Surgeon	M	40-50	• Vice professor specializing in pancreatic cancer
17	Urinary Surgeon	M	50-60	• Professor specializing in urinary cancer
18	Urinary Surgeon	M	30-40	• Attending physician specializing in urinary cancer
19	Breast Surgeon	M	40-50	• Vice professor specializing in breast cancer
20	Hepatic Surgeon	M	40-50	• Vice professor specializing in hepatic cancer<br>• Expert in neoadjuvant therapy
21	Oncologist	F	50-60	• Professor specializing in thoracic cancer chemotherapy
22	Oncologist	M	40-50	• Attending physician in the collaborating secondary hospital that took part of the neoadjuvant chemotherapy for our hospital’s patients
23	Anesthesiologist	F	40-50	• Vice professor<br>• Expert in ERAS

				• Collaborated with the departments of anesthesia and rehabilitation to promote prehabilitation for years<br>• Worked in preoperative anesthesia evaluation and optimization outpatient clinics
24	Anesthesiologist	F	30-40	• Attending physician<br>• Conducted research on prehabilitation
25	Anesthesiologist	F	40-50	• Vice professor<br>• Worked in preoperative anesthesia evaluation and optimization outpatient clinics
26	Rehabilitation physician	F	30-40	• Attending physician<br>• Collaborated with the departments of anesthesia and thoracic surgery to promote prehabilitation for years
27	Physical Therapist	F	30-40	• Provided exercise guidance for both outpatient and hospitalized patients
28	Nurse	F	30-40	• Supervised and recorded the exercise practice of hospitalized patients

Abbreviations: M, male; F, female; ERAS, enhanced recovery after surgery

Supplements S4: Quotations

Theme	Subtheme	Quotations
**Capacity**	Surgeon's ability to gain trust and adherence from patients	• Once a patient sees clear results from neoadjuvant therapy, such as symptom relief, they will have a strong desire for surgery and will be more willing to cooperate with doctors. After all, the effective results increase their trust. During the 2-3 months of neoadjuvant therapy, having something to do can help alleviate anxiety. (participant 8, surgeon)<br>• We've been cooperating with the thoracic surgeons for many years. In the beginning, patients didn't understand why they needed exercise prehabilitation. We had to spend a lot of time explaining it. Later, the thoracic surgeons began educating patients about the importance of prehabilitation, and things became earlier, since patients tended to trust their surgeons more. (participant 26, rehabilitation physician)<br>• My surgeon is the person I trust the most. If he told me: "You must exercise to a certain level, or we won't proceed with the surgery," I definitely wouldn't be this lazy. After all, no one wants to risk their life. (participant 3, patient)
	Physician's lack of knowledge in exercise prehabilitation	• I am not very familiar with some specialized exercises, so I'm unable to provide specific instructions, such as pelvic floor exercises for colorectal or gynecological surgery, and specific muscle exercises for orthopedic surgery. (participant 23, anesthesiologist)<br>• Some patients' conditions are quite complex—some have had strokes, some have joint issues, and others

		have deep vein thrombosis in their lower limbs. (participant 11, surgeon)<br>• I generally advise patients to exercise according to their capabilities and to understand their own limits. I encourage them to exercise but do not provide very specific instructions, as my knowledge in these areas is limited. (participant 22, oncologist)
	Physician’s lack of time and energy for patient education in prehabilitation	• Generally, we focus on performing surgeries well and minimizing the risk of complications. We don’t have time to address exercise and nutrition in detail. (participant 12, surgeon)<br>• Each patient only has a few minutes during their outpatient visit, so there's no time for in-depth discussions on exercise. (participant 13, surgeon)
	Patient's prior commitment to regular exercise	• I have been doing farm work since I was young. I can't stay idle. Even after getting sick, walking for one hour a day is not a difficult task for me. (participant 1, patient)<br>• Some patients used to exercise regularly before getting sick, and they are eager to return to their previous state. As soon as they start feeling better after completing chemotherapy, they often ask questions like, “When can I start lifting weights again?”(participant 12, surgeon)
**Opportunity**	Lack of standardized referral process to the rehabilitation clinic	• Doctors in each department are busy, and if they have to handle it themselves and don't know whom to refer the patient to, they may be reluctant to do it. (participant 26, rehabilitation physician)<br>• I don’t feel confident in providing patients with specific exercise recommendations, but I'm unsure

		which department to turn to for assistance. (participant 20, surgeon)<br>• Most patients have the rehabilitation department involved after being admitted to the hospital for surgery. It's rare for them to get involved in outpatient clinics during neoadjuvant therapy. Unfortunately, the preoperative hospital stay is relatively short, so there's only 2-4 days for practice. (participant 27, physical therapist)
	Lack of follow-up and supervision for patients	• Many patients are from other provinces or rural areas and are unwilling to come all the way to Beijing just for exercise rehabilitation guidance. Even if they visit the rehabilitation outpatient clinic, it's usually on the same day as their surgical or chemotherapy appointments, meaning they often receive in-person guidance only once — and one session is often not enough. (participant 26, rehabilitation physician)<br>• After completing a chemotherapy cycle, patients typically start to improve gradually within 3-5 days, with the next cycle beginning after 21 days. During this inter-cycle period, they are generally comfortable, almost back to a normal state. However, during this phase, when they are physically at their best, there is no doctor overseeing their care or encouraging them to do exercise. They are simply resting. (participant 6, surgeon)
	Conflicts with traditional cultural belief	• There are still many misconceptions among Chinese patients. Once they fall ill, they stop doing not only exercise but even basic household chores, opting for complete rest instead. Patients tend to focus more on "survival" and have limited expectations about

		returning to normal life after surgery. (participant 8, surgeon)<br>• Many patients are afraid to move after getting sick, and we need to provide a sense of reassurance for them. (participant 28, nurse)<br>• After getting sick, I still want to exercise, but I hesitate because people around you constantly warn: “Don’t push yourself too hard—what if something goes wrong?” (participant 2, patient)
	Lack of support from insurance policies	• Prehabilitation-related costs are not covered by insurance; only postoperative rehabilitation is reimbursed. Some patients are unwilling to pay out of pocket. (participant 26, rehabilitation physician)<br>• Costs not covered by insurance are often questioned. Patients may feel that out-of-pocket expenses are unnecessary and may doubt the doctor's intentions. (participant 7, surgeon)<br>• We are willing to pay some costs for prehabilitation, but not too much. (participant 5, a patient’ relative)
**Motivation**	Lack of awareness about the benefits of exercise	• In my view, the importance is ranked as follows: nutrition, psychology, and then exercise. (participant 17, surgeon)<br>• We focus more on anorectal, sexual, and urinary functions, often limiting our attention to functions related to the surgical area, and rarely consider overall physical function. (participant 14, surgeon)<br>• If oncologists understand that exercise can improve patients' conditions and make them more tolerant of chemotherapy, and if surgeons realize that better physical fitness can allow for more thorough surgeries,

		they will pay more attention to it. They have not experienced the difference between exercising and not exercising. (participant 26, rehabilitation physician)
	Concerns about patients' exercise capabilities	• Patients who come to me are usually quite weak, often suffering from anemia and poor nutrition, making exercise challenging. Therefore, I don’t emphasize it too much. (participant 13, surgeon)<br>• For those undergoing intensive, being able to walk on their own by the third week is a challenge for them. Most patients experience nausea at home, struggle to get out of bed, and are not in a condition to engage in physical exercise. (participant 9, surgeon)<br>• I rarely encourage them to exercise actively, mainly because they are often in very poor condition. If they can stay alive, eat, and sleep, that’s already considered an achievement. Exercise is not a priority. (participant 10, surgeon)
	Lack of awareness about patients' insufficient physical fitness	• We often believe our patients typically do not have significant issues with fitness; however, reduced physical strength from chemotherapy are generally not visible. (participant 16, surgeon)<br>• Including a physical fitness assessment as part of preoperative evaluations would be very beneficial, as otherwise, these issues might go unnoticed. (participant 25, anesthesiologist)
**Behavior**	Physician education on exercise prehabilitation	• Doctors have a significant impact on patients. It’s more effective to educate doctors first than to educate patients. While we don't need to know the details, it's necessary to understand the basic concepts. (participant 21, oncologist)

		• I knew little about prehabilitation before, but after this interview, I would like to learn more about it. (participant 16, surgeon)
	Multidisciplinary team clinic	• I recommend to establish a prehabilitation multidisciplinary clinic that comprehensively addresses all preoperative preparations, including smoking cessation, personalized exercise and nutrition plans, psychological support, medication adjustments, and blood sugar and blood pressure management. The goal is to streamline care and prevent patients from having to visit multiple clinics separately. (participant 11, surgeon)
	Online remote exercise supervision	• We are experimenting with remote guidance, including question & answer, a video library of exercise demonstrations, and check-ins. Initially, this was for research purposes. We created a group where patients can give us feedback, including their physical reactions and heart rate data. (participant 26, rehabilitation physician; participant 24, anesthesiologist) • Guidance can be provided not only face-to-face but also through printed materials or digital content accessible by scanning a QR code. (participant 15, surgeon)

	Simplified plan and measurable goals	• Simple tasks, such as the 6-minute walk test, can still provide valuable insights into a patient's physical fitness and prognosis. The simpler, the better—no need to complicate things. (Participant 23, anesthesiologist)<br>• We ask patients to wear a fitness watch and keep their heart rate within a specified range. (participant 6, surgeon)<br>• Our ward nurses assist by using a checklist to track the patient's exercise progress, indicating what has been completed and what still needs to be done. (participant 28, nurse)
